# Sudden cardiac arrest on the field of play: turning tragedy into a survivable event

**DOI:** 10.1007/s12471-018-1084-6

**Published:** 2018-02-06

**Authors:** David M. Siebert, Jonathan A. Drezner

**Affiliations:** Department of Family Medicine, UW Medicine Center for Sports Cardiology, University of Washington, 98195 Seattle, WA USA

**Keywords:** Automated external defibrillator, Emergency action plan, Sudden cardiac death, Athlete, Collapse, Exercise

## Abstract

Sudden cardiac arrest remains the leading cause of death in exercising athletes, and recent studies have shown that it occurs more frequently than historical estimates. While out-of-hospital cardiac arrest often proves fatal, advance preparation can improve outcomes and the chance of survival. First responders to a collapsed athlete on the field of play may include team medical personnel, coaches, other athletes, officials, venue staff, emergency medical services personnel, or lay bystanders. Prompt and accurate recognition of sudden cardiac arrest, a comprehensive and rehearsed emergency action plan, early cardiopulmonary resuscitation, and immediate access to and use of an automated external defibrillator are each pivotal links in the chain of survival. This review summarises the components of an effective emergency action plan, highlights the critical role of automated external defibrillators, and reviews the diagnosis and management of sudden cardiac arrest on the field of play.

## Introduction

**Figure:**
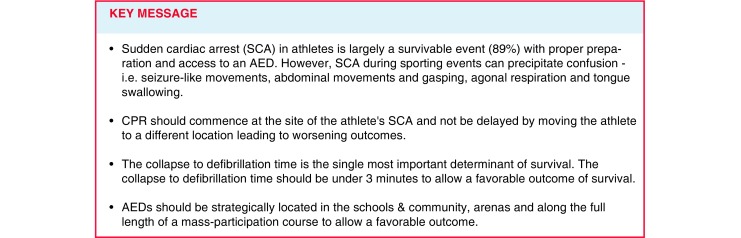

The sudden collapse and death of a young athlete on the field of play is a tragic event with a profound impact on the local community. Sudden cardiac arrest (SCA) is the leading cause of sudden death in athletes, accounting for 75% of all deaths during exercise and sport [1]. Recent epidemiological data have shown that SCA strikes competitive athletes more frequently than historical estimates. For example, annual risks have been shown to be 2.3:100,000 in Italian athletes [2] and 1–3:100,000 in professional soccer players [3]. The Fédération Internationale de Football Association (FIFA) has begun a registry to study all cases of soccer-related SCA to better understand the extent of the problem [4]. In the United States, male athletes, African/Afro-Caribbean athletes and basketball, American football and soccer athletes are at highest risk [1, 5–12]. The cardiovascular diseases and conditions that precipitate SCA comprise a heterogeneous group of disorders that usually exhibit no symptoms or warning signs prior to the sentinel event of SCA [1, 12]. As a result, preparticipation cardiovascular screening is challenging and the optimal strategy to detect athletes with disorders at risk of sudden death remains controversial [13–15]. Thus, medical personnel responsible for athlete safety must be prepared to recognise, respond to, and effectively treat SCA.

## Emergency response planning for sudden cardiac arrest

SCA is a life-threatening event and requires a prompt and coordinated medical response. Preparation and rehearsal of an emergency action plan (EAP) are essential to decrease the chance of a catastrophic outcome. Guidelines for the development and implementation of an EAP have been previously outlined [15–19] and are broadly supported by the sports and medical communities. A comprehensive EAP includes training of likely first responders to a collapsed athlete, including on-site medical and club personnel, as well as access to an automated external defibrillator (AED), emergency medical services, and advanced care facilities (Tab. 1). A thorough understanding of emergency preparedness guidelines is imperative to allow for the efficient execution of the medical response to SCA.

**Table 1:** Essential components of an emergency action plan for sudden cardiac arrest

*1. Personnel training*: potential first responders should be trained in CPR and the use of an AED
*2. Site or venue specificity*: EAPs should be venue specific and clearly demark the location of AEDs and transportation routes for EMS
*3. Means of efficient communication*: reliable communication for the on-site medical team and to activate EMS
*4. Immediate AED access*: retrieval and use of an AED within 3 min of collapse
*5. Regular review and rehearsal:* the EAP for SCA should be practiced at least annually among anticipated first responders
*6. Advanced cardiac care facility*: proficient in advanced life support, cardiac care, and induced hypothermia
*7. Reset, debriefing, and reporting*: replacement of emergency equipment and personnel before play resumes

*CPR* cardiopulmonary resuscitation, *EAP* emergency action plan, *SCA* sudden cardiac arrest,* EMS* emergency medical services, *AED* automated external defibrillator

### Components of an emergency action plan

According to best-practice recommendations [15–19], an effective cardiac EAP includes the following:

1. *Personnel training*: All potential first responders, including team medical staff (athletic trainers, physiotherapists, and physicians), coaches, and appropriate school or venue staff, should be trained in cardiopulmonary resuscitation (CPR) and the use of an AED. An emphasis on the prompt and accurate recognition of SCA is an essential part of this training. SCA should be assumed in any collapsed and unresponsive athlete and the EAP activated [17].
2. *Site or venue specificity*: Each EAP should be carefully designed for the specific site or venue at which it will be executed. It should clearly depict the location of the nearest AED, provide a facility map, and list emergency contact phone numbers and relevant street address. Directions to guide emergency medical service providers to the location of SCA and previously defined venue entry and exit points are recommended, especially in the case of large stadiums, parks, or complexes.
3. *Means of efficient communication*: Reliable methods of communication between on-site personnel and emergency medical services should be established and tested prior to competition. The reliability of cellular phone service, especially at events with high spectator attendance, merits consideration.
4. *Immediate AED access*: Access to an AED must be prompt, direct, and reliable. AEDs should be strategically placed to allow retrieval, application, and shock deployment within 3 min of collapse [16]. Attention should be given to potential physical barriers to AED retrieval, such as doors or gates. AEDs can be used by any bystander, and public access to AEDs has clearly improved survival from SCA [20–24]. Proper signage should indicate to responders the location of an AED and the devices should never be locked away or inaccessible, especially during training and competitions. AEDs should be maintained according to manufacturer recommendations. Prior to each scheduled event, a ‘readiness’ check is also recommended to confirm the presence of the AED in the designated location and to inspect the indicator light to ensure proper functioning.
5. *Regular review and rehearsal*: All personnel potentially involved in the care of an athlete with SCA should review and rehearse the EAP at least once per year. Roles for each rescuer should be clearly defined, and a mock SCA scenario is recommended to practice a coordinated response. Prior to each competition, brief review of the EAP by home and visiting medical staffs is also recommended. Adjustment of the EAP may be necessary when personnel or community resources change.
6. *Advanced cardiac care facility*: The nearest advanced cardiac care facility should be integrated within the EAP and defined as the preferred receiving hospital for a transferred athlete. This facility should have critical care and advanced life support capabilities and, if possible, proficiency in induced hypothermia, which may improve outcomes after cases of resuscitated SCA [25]. The pending transfer of an athlete following SCA should be communicated with the receiving cardiac care facility as soon as possible.
7. *Reset, debriefing, and reporting*: If play is to continue following transfer of an athlete, a reset of the EAP and repositioning of related personnel is necessary prior to resuming competition. This reset may include the restoration of equipment to its original location and the replacement or reassignment of personnel roles and necessary emergency medical services. AED electrodes should be replaced, and batteries and operational status retested. A debriefing session should occur for all rescuers involved in an athlete’s resuscitation to review and document the medical response. Counselling and psychological support should be provided to rescuers and teammates as needed. Reporting of the event to appropriate authorities can assist research efforts and may improve future responses and the treatment of SCA.

## Value, role, and placement of automated external defibrillators

Historical survival rates to hospital discharge following out-of-hospital cardiac arrest in the general population have been poor, as low as 7.6% [26]. The time from collapse to defibrillation following the onset of SCA is the single most important determinant of survival [27]. Modern public access AED programmes have yielded higher survival rates of 19.7 to 44% [20–24]. A meta-analysis reported a median survival rate of 40% with public access to AEDs [20].

Access to early defibrillation also improves survival in young athletes with SCA. In a 2-year prospective observational study of 2,149 high school campuses, student-athletes with SCA survived 89 % of the time if prompt CPR and defibrillation were provided [28]. Survival was also higher if an on-site AED was used versus supplied by responding emergency medical services [28].

AEDs should be strategically located to allow a collapse-to-shock time of under 3 min. Epidemiological data can also be considered to prioritise specific locations. Studies demonstrate that the incidence of SCA in United States athletes is higher in certain subgroups, specifically male athletes, African/Afro-Caribbean athletes and American football, basketball, and soccer players [1, 5–12]. In schools and community complexes, the gymnasium is the most likely site for SCA and offers a central location for AED placement that services a number of sporting activities and large public gatherings [13, 28]. The size of the venue and number of anticipated event spectators may also influence AED placement, as on-site medical personnel may be best positioned to assist a collapsed spectator prior to emergency medical services arrival. Guidelines are available regarding the number and location of AEDs in large sporting venues and arenas [29–31].

For large mass-participation events such as marathons and triathlons, emergency care is ideally provided along the full length of the course. However, SCA during long-distance runs occurs more frequently in the late stages of the race [32, 33]. These data should be considered when making resource allocation decisions, such as the pre-race distribution of AEDs, placement of roaming or bicycle emergency medical service responders, and organisation of finish line ‘catchers’ and medical volunteers.

## Recognising sudden cardiac arrest

Exercise can increase the risk of SCA in athletes with underlying structural or electrical cardiac disorders [2]. Any athlete who collapses without obvious head trauma and is unresponsive on the field of play should be assumed to be in SCA until proven otherwise. Blunt trauma to the chest causing collapse, usually from a firm projectile, is consistent with commotio cordis and should also be treated as SCA.

In over 50% of cases, athletes with SCA demonstrate brief myoclonic or seizure-like activity following collapse [9, 34]. Thus, SCA should not be mistaken for an epileptic seizure, which may delay the initiation of CPR, retrieval of an AED, or even discourage these critical steps altogether. Athletes with SCA may also display abdominal movements or gasps that can be mistaken for normal breathing. In this scenario, rescuers may misinterpret these agonal respirations and delay the recognition and treatment of SCA. To avoid life-threatening delays in resuscitation, brief seizure-like activity and/or agonal respirations should be assumed due to SCA in a collapsed and unresponsive athlete, and initial management steps for SCA should be taken immediately, unless a non-cardiac cause of collapse is clearly determined. Furthermore, the myth of a need to prevent ‘tongue swallowing’ after collapse has been shown to delay bystander chest compressions [35] and should be discouraged as an unnecessary procedure during resuscitation.

## Management of sudden cardiac arrest

Management of SCA on the field of play starts with making a prompt diagnosis. Once SCA is suspected or confirmed, the EAP should be initiated expeditiously. If multiple rescuers are available, one rescuer should begin CPR while another rescuer contacts emergency medical services and a third retrieves the closest AED if one is not already on the field of play. If there is only one rescuer, emergency medical services should be activated and then an AED obtained if readily available, with the rescuer returning to the victim to apply the AED and begin CPR.

CPR should commence at the site of athlete arrest, beginning with chest compressions in the case of a witnessed collapse. Moving the athlete to a different location may delay the resuscitation and could lead to worse outcomes [36]. Medical personnel should be prepared to quickly remove protective padding, clothing, or other equipment that may impede the initiation of CPR; this may require specialised tools or equipment. Chest compressions should be at least five to six centimetres or two inches in depth, allow complete chest recoil, and occur at a rate of 100–120 per minute [37, 38]. CPR should continue uninterrupted until an AED is applied and begins to analyse the athlete’s cardiac rhythm.

Rescuers should exercise considerable caution in excluding SCA in a collapsed and unresponsive athlete. Since the vast majority of these athletes are in ventricular fibrillation, an AED should be applied and turned on for rhythm analysis as soon as possible. AEDs are extremely accurate in recommending a shock when ventricular fibrillation or rapid ventricular tachycardia is present. If defibrillation is provided, chest compressions should resume immediately after shock delivery. If a shock is not advised, CPR should be continued until advanced life support providers take over or the victim starts to move. High-quality CPR should continue until return of spontaneous circulation is achieved or death is declared at a receiving facility [37, 38].

## Conclusions

SCA in athletes is a life-threatening event that can precipitate confusion during sporting events. Development and rehearsal of a site-specific EAP is essential to ensure an efficient response and the best chance of survival. With prompt and accurate recognition of SCA, provision of high quality CPR, and access to early defibrillation via strategically placed AEDs, SCA in an exercising athlete is largely a survivable event.
